# *NTRK* Fusions in 1113 Solid Tumors in a Single Institution

**DOI:** 10.3390/diagnostics12061450

**Published:** 2022-06-13

**Authors:** Heejin Bang, Mi-Sook Lee, Minjung Sung, Juyoung Choi, Sungbin An, Seok-Hyung Kim, Seung Eun Lee, Yoon-La Choi

**Affiliations:** 1 Department of Pathology, Konkuk University Medical Center, Konkuk University School of Medicine, Seoul 05030, Korea; mars-21@hanmail.net; 2 Laboratory of Theranotics and Molecular Pathology, Samsung Medical Center, Sungkyunkwan University School of Medicine, Seoul 06351, Korea; misook98@gmail.com (M.-S.L.); mjsung0111@gmail.com (M.S.); juyoung.choi92@gmail.com (J.C.); been9312@naver.com (S.A.); 3 Department of Health Sciences and Technology, SAIHST, Sungkyunkwan University, Seoul 06351, Korea; 4 Department of Pathology and Translational Genomics, Samsung Medical Center, Sungkyunkwan University School of Medicine, Seoul 06351, Korea; platoshkim@daum.net

**Keywords:** *NTRK* fusion, TRK immunohistochemistry, next-generation sequencing, TRK inhibitors, lung cancer, colon cancer, inflammatory myofibroblastic tumor

## Abstract

**Simple Summary:**

Recently, there has been increasing interest in identifying *NTRK* fusions in various tumors, as they are therapeutically targetable driver mutations. In tumor types with low-frequency *NTRK* fusions, recent recommendations on *NTRK* testing recommend pan-Trk immunohistochemistry (IHC) as the initial screening test to validate pan-Trk expression cases with next- generation sequencing (NGS) assays. This retrospective study was conducted on 1113 solid tumor samples (510 non-small cell lung cancers, 503 colorectal cancers, and 100 inflammatory myofibroblastic tumors) to evaluate using pan-Trk IHC assay, and TRK expression cases were followed by validation with NGS. We investigated the accuracy of an IHC assay in detecting *NTRK* fusions and characterizing the clinicopathological and molecular features of *NTRK*-rearranged common tumors. Despite its rarity, this study confirms the importance of identifying potential target groups based on the pathological and immunohistochemical characteristics of *NTRK* fusion-driven solid tumors for effective targeted therapy.

**Abstract:**

Most *NTRK* fusions occur at very low frequencies in various common cancers. Recent recommendations on *NTRK* testing recommend immunohistochemistry (IHC) as the initial test for tumor types with a low frequency of *NTRK* fusions. This study investigated the accuracy of an IHC assay to detect *NTRK* fusions and characterize the clinicopathological and molecular features of *NTRK*-rearranged tumors. This retrospective study was conducted on 1113 solid tumor samples known to harbor no oncogenic driver alterations, including 510 non-small cell lung cancers (NSCLC), 503 colorectal cancers (CRC), and 79 inflammatory myofibroblastic tumors (IMT). Additionally, 21 ALK expression-positive cases were included. TRK expression was evaluated using a pan-Trk IHC assay, and positive cases were validated using NGS. TRK expression was observed in three NSCLCs (0.6%), six CRCs (1.2%), and six IMTs (6%). *NTRK* fusions were finally detected in two NSCLCs (0.4%), six CRCs (1.2%), and one IMT (1%). In NSCLC and CRC, the majority of *NTRK* fusions were readily discernible due to diffuse moderate-to-strong cytoplasmic staining on pan-Trk IHC. In IMT, focal weak nuclear staining indicated the presence of *NTRK* fusion. Therefore, the utility of pan-Trk IHC should be assessed considering that the difference in performance depends on tumor type.

## 1. Introduction

Members of the tyrosine receptor kinase (TRK) family bind to neurotrophins and affect neuronal differentiation and survival, thereby playing important roles in the nervous system. *NTRK*s, including *NTRK1/2/3*, encode tropomyosin receptor kinase A/B/C (TRKA/B/C), respectively [1,2]. *NTRK* fusion can cause constitutive activation of TRK receptors and overexpression of TRK proteins, which can lead to oncogenesis in various types of cancers [1,3]. 

Recently, there is increasing interest in identifying *NTRK* fusions in various tumors, as they are therapeutically targetable driver mutations. Two TRK inhibitors have received FDA therapeutic approval for the treatment of *NTRK* fusion-positive tumors [4]. Entrectinib (Genetech, Roche) was the first drug developed against *NTRK* fusions, which also targets *ALK* and *ROS1* fusion proteins, and was designated as an orphan drug for *NTRK* fusion-positive non-small cell lung cancer (NSCLC) and colorectal cancer (CRC) by the FDA in 2015 [5,6]. Larotrectinib (VITRAKVI, Loxo Oncology Inc., Bayer) is highly specific for *NTRK* fusions and was designated as a breakthrough therapy for *NTRK* fusion-positive solid tumors in 2016 [3]. In 2018, the FDA accepted a new drug application and granted a priority review for larotrectinib in the treatment of adult and pediatric patients with locally advanced or metastatic solid tumors harboring an *NTRK* fusion regardless of tumor type [7].

Tumor types where *NTRK* fusions are characteristic or even considered pathognomonic, such as secretory breast carcinoma and secretory carcinoma of the salivary gland, infantile fibrosarcoma, and congenital mesoblastic nephroma, are very rare [8,9,10,11]. Conversely, the majority of *NTRK* fusions occur at very low frequencies, with an average rate of 0.5–1% in a variety of common cancers—such as lung adenocarcinoma, colorectal and malignant melanoma, and soft tissue sarcoma [12,13,14,15]. However, these common cancer types contribute to most patients with *NTRK* fusions. 

Therefore, it is important to identify patients who could benefit from TRK inhibitor therapy using reliable and cost-effective techniques for common cancer types that rarely harbor *NTRK* fusions. In tumor types with a low frequency of *NTRK* fusions, recent *NTRK* testing recommendations suggesting using pan-Trk immunohistochemistry as a screening tool to identify cases for definitive *NTRK* fusion detection by NGS assay. RNA-based targeted NGS assays to detect NTRK fusions can accurately characterize fusion transcripts if sufficient RNA of adequate quality is available [16].

In this study, to uncover the *NTRK* fusion frequency in the South Korean population with NSCLC, CRC, and inflammatory myofibroblastic tumors (IMT), we performed a pan-Trk IHC assay and confirmed the pan-Trk-positive samples with NGS assays. Furthermore, we investigated the accuracy of an IHC assay to detect *NTRK* fusions and characterize the clinicopathological and molecular features of *NTRK*-rearranged tumors.

## 2. Materials and Methods

### 2.1. Case Selection

A total of 1113 patients with solid tumors who underwent surgical resection or biopsy at Samsung Medical Center between January 2010 and April 2020 were selected. These included 510 NSCLCs, 503 CRCs, and 100 IMTs. All cases were pathologically confirmed. Patients with NSCLC and CRC were excluded if they had known driver mutations, such as *ALK*, *ROS1*, *BRAF*, and *EGFR* mutations in NSCLC and *KRAS*, *NRAS*, and *EGFR* mutations in CRC. Of the total 503 CRCs, 333 cases had microsatellite instability (MSI) analysis information, of which 14 cases were MSI-H (high-level MSI). The IMT included 21 ALK expression-positive cases and 79 ALK expression-negative cases. *EGFR* alteration was detected by either real-time PCR with PNA-clamping methods, direct sequencing, or both methods. For the *ALK* fusion, ALK IHC or fluorescence in situ hybridization (FISH) was performed, and *ROS1* fusion was detected by RT-PCR. *BRAF*, *KRAS*, and *NRAS* alterations were detected by RT-PCR or NGS assay. Patients with inadequate tumor specimens for molecular analysis were excluded. Clinical data on sex, age at surgery, smoking history, tumor histology, and tumor size were extracted from electronic medical records. This study was reviewed and approved by the Institutional Review Board of Samsung Medical Center (#2020-04-105-003).

### 2.2. Tissue Microarrays (TMA)

TMAs were constructed to include two 3 mm cores of representative tumors in formalin-fixed paraffin-embedded (FFPE) tissues from 1113 cases. IHC using TMAs was reviewed by two pathologists (HB and SEL).

### 2.3. IHC Assay

The FFPE samples used in this study were tissues from NSCLC, CRC, and IMT patients diagnosed between 2013 and 2020. FFPE TMA blocks were cut into 4 μm thick sections and placed on slides. We used a commercially available pan-Trk assay (rabbit monoclonal antibody, clone EPR17341 assay, ready to use (RTU), Roche, Ventana, Oro Valley, AZ, USA) to screen for TRK expression in FFPE specimens. In the case of the pan-Trk IHC assay, there is no scoring algorithm or criteria for determining IHC positivity. Tumors were considered positive when tumor cells exhibited staining at any intensity if ≥1% of tumor cells. In addition, different subcellular staining patterns (nuclear, cytoplasmic, membranous, etc.) were considered positive.

### 2.4. NGS Analysis

A total of 15 IHC-positive cases with available FFPE material were analyzed by NGS to confirm *NTRK* fusion status and identify possible fusion partners. NGS was conducted using the TruSight^TM^ Oncology (TSO) 500 assay (Illumina) according to the manufacturer’s recommendations. The positive samples were further validated by an additional hybridization capture-based targeted RNA panel (SOLIDaccuTest RNA), which includes all exons of *NTRK1/2/3*.

## 3. Results

### 3.1. Prevalence of NTRK Fusions in 1113 Solid Tumors

Of the 1113 solid tumor samples screened with pan-Trk IHC, 15 cases (1.3% of the entire cohort) showed TRK expression. RNA-based NGS assay identified 15 cases, of which nine (0.8% of the entire cohort) had an *NTRK* fusion. The clinicopathological characteristics of the 15 TRK expression cases (three NSCLCs, six CRCs, and six IMTs) are summarized in Table 1.

The pan-Trk immunohistochemical and molecular characteristics of the 15 TRK expression cases are summarized in Table 2. *NTRK1* fusion was the most common (*n* = 6), followed by *NTRK3* (*n* = 3). Fusion was not observed in *NTRK2*. Two *NTRK*s were involved in the fusion with five different partner genes: *TPM3–NTRK1*, *LMNA–NTRK1*, *CD74–NTRK1*, *ETV6–NTRK3*, and *SQSTM1–NTRK3.* These five types of *NTRK* fusion have been previously reported in various tumors. Strong and uniform expression with pan-Trk IHC identified 5/6 *NTRK1* fused cases, and all *NTRK3* fusion cases showed moderate staining intensity. Of the 15 TRK expression positive cases, six *NTRK* fusion-negative, namely false-positive pan-Trk IHC cases, showed weak and moderate staining intensity but not strong staining intensity. The subcellular distribution of immunohistochemical staining differed depending on the fusion partner. 

### 3.2. Non-Small Cell Lung Cancer

Of the 510 patients diagnosed with NSCLC, 341 (66.9%) were male, and the median age was 64.6 years (range, 32–84 years). 

**Figure 1:**
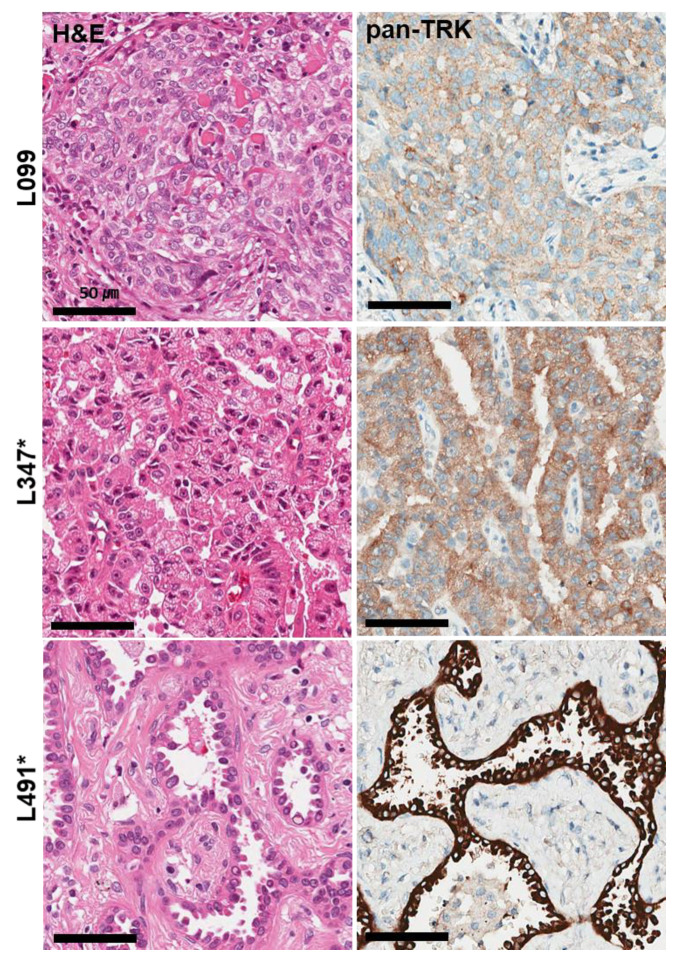
Histological and immunohistochemical findings of three TRK expression cases observed in 510 NSCLC samples. Pan-TrK IHC with moderate cytoplasmic and membranous staining in NSCLC with a *SQSTM1-NTRK3* fusion (L347 case). Pan-TrK IHC with strong cytoplasmic and membranous staining in NSCLC with a *CD74-NTRK1* fusion (L491 case). * Confirmed cases of *NTRK* gene fusions in NGS assays.

All cases were immunohistochemically analyzed using VENTANA pan-Trk IHC, and TRK expression was observed in 3 of 510 cases (0.6%); (L099, L347, L491) (Figure 1). Pan-Trk IHC staining showed cytoplasmic and membranous staining in the tumor cells of all three cases but with different intensity (1–3). The three positive cases were further validated using RNA-based targeted NGS assay (TSO500). Immunohistochemical analysis of pan-Trk was concordant with the TSO500 assay in two of three cases. Finally, two NSCLC cases harbored *NTRK* fusion among 510 NSCLCs (0.4%). In the L347 and L491 cases, *NTRK* fusion genes were detected with *SQSTM1–NTRK3* and *CD74–NTRK1*, respectively. Non-*NTRK*-rearranged cases showed cytoplasmic staining with weak to moderate intensity for pan-Trk antibodies in IHC. 

The average age of *NTRK*-rearranged NSCLC patients was 48 years, which was younger than that of all NSCLC patients (64.6 years old). The two *NTRK*-rearranged NSCLCs were moderately differentiated adenocarcinomas showing papillary and acinar patterns in a 42-year-old male and a 54-year-old female, respectively. 

### 3.3. Colorectal Cancer

In 503 patients with CRC, the median age at diagnosis was 58.2 years (range, 17–87 years), and 299 (59.4%) were male. 

**Figure 2:**
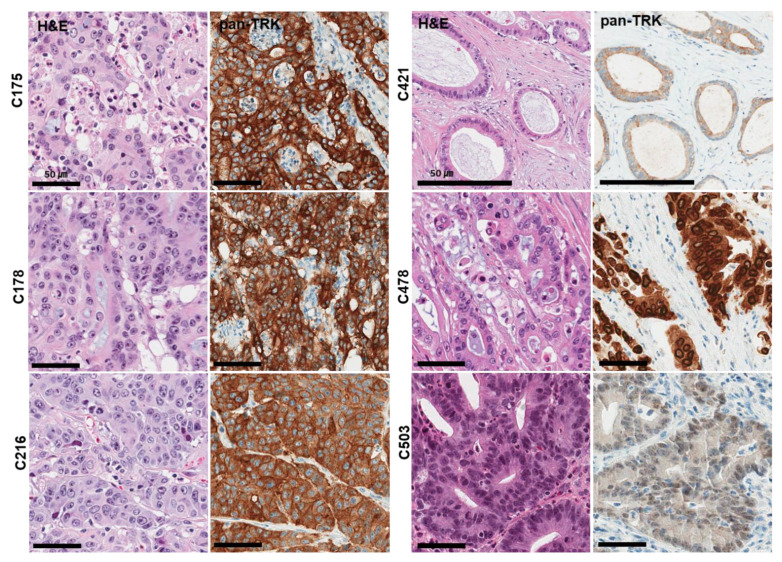
Histological and immunohistochemical findings of six TRK expression cases observed in 503 CRC samples (Confirmed cases of *NTRK* gene fusions in NGS assays). Pan-TrK IHC with strong cytoplasmic and membranous staining in CRC with a *TPM3-NTRK1* fusion (C175, C178, C216 case). Pan-TrK IHC with weak to moderate cytoplasmic and membranous staining in CRC with a *TPM3-NTRK1* fusion (C421 case). Pan-TrK IHC with strong nuclear membranous staining in CRC with a *LMNA-NTRK1* fusion (C478 case). Pan-TrK IHC with moderate nuclear staining in CRC with an *ETV6-NTRK3* fusion (C503 case).

From pan-Trk IHC assays, a total of six cases (1.2%) were found to express TRK proteins (C175, C178, C216, C421, C478, C503) (Figure 2). *NTRK* fusions were detected in all five cases. (1.2%). Three partner genes were identified: four cases of *TPM3–NTRK1* and one case each of *LMNA–NTRK1* and *ETV6–NTRK3*. Immunohistochemical analysis of pan-Trk was concordant with the TSO500 assay. Pan-Trk IHC staining showed cytoplasmic and membranous staining in the four *TPM3–NTRK1* cases, nuclear membranous staining in the *LMNA–NTRK1* case, and nuclear staining in the *ETV6–NTRK3* case (Table 2). Immunoreactivity for TRK was easily identifiable, as the majority of positive CRC cases showed strong, uniform intensity staining. 

Interestingly, all six *NTRK*-rearranged CRCs were MSI-H tumors. The average age of *NTRK*-rearranged CRC patients was 69.8 years old, which was older than that of all CRC patients (58.2 years old). Histologically, all *NTRK* fusion cases were adenocarcinomas, and no characteristic histological features were identified.

### 3.4. Inflammatory Myofibroblastic Tumor

**Figure 3:**
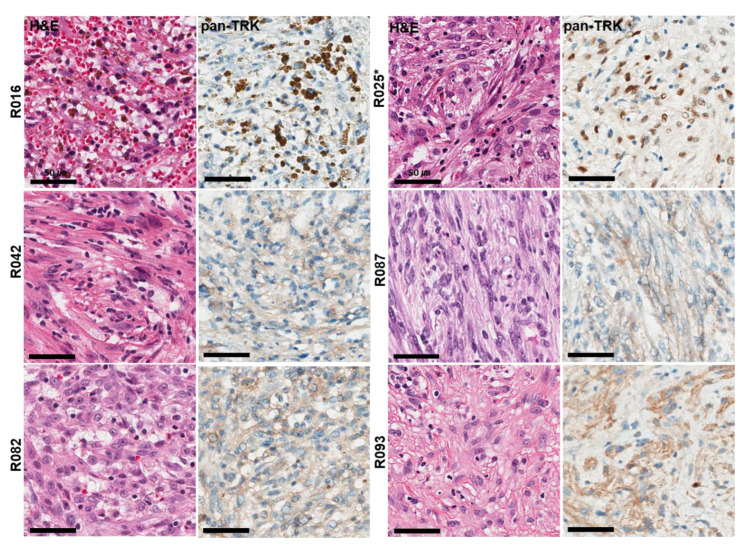
Histological and immunohistochemical findings of six TRK expression cases observed in 100 IMT samples. Pan-TrK IHC with moderate nuclear staining in IMT with an *ETV6-NTRK3* fusion (R025 case). * Confirmed cases of *NTRK* gene fusions in NGS assays.

In 100 patients with IMT, the median age was 45.3 years (range, 1–81 years), and 43 (43.0%) were female. Six cases (6%) expressed TRK in pan-Trk IHC assays, including five cases of weak to moderate cytoplasmic staining and one case of moderate nuclear staining (Figure 3). 

Of the six cases of TRK-expressing IMT, *NTRK* fusion was detected in only one case (R025), which showed moderate nuclear staining in pan-Trk IHC assays. *NTRK* fusion transcripts were identified with *ETV6–NTRK3* (exon 5 of *ETV6* fused with exon 15 of *NTRK3*) using NGS. Non-*NTRK* rearranged cases showed cytoplasmic but not nuclear staining for pan-Trk antibody. Finally, only one case harbored *NTRK* fusion in 100 IMTs (1%). 

The *NTRK*-rearranged IMT was identified in a 41-year-old female who presented with a 5.5 cm solitary, well-defined lung mass with no metastatic lesions identified at presentation. Histologically, two patterns, including a cellular area with cytologically bland spindle cells and a prominent lymphoplasma cells infiltrate and a less cellular myxoid area with spindled myofibroblasts showing vesicular nuclei, small nucleoli, and eosinophilic cytoplasm, were observed in the R025 case. TRK immunoreactivity showed heterogeneous expression and easily identifiable moderate nuclear staining intensity in less cellular myxoid areas.

## 4. Discussion

Identifying patients harboring *NTRK* fusions is very important in routine diagnostic practice. It is critical to have a good discovery strategy because the incidence of *NTRK* fusions is extremely low. First, it is essential to verify the prevalence of *NTRK* fusion in common solid tumors reported in other studies. Common cancer types, such as lung and colon cancer, harbor *NTRK* fusions with a prevalence <1%. In this study, the prevalence of *NTRK* fusion in NSCLC and CRC was 0.4% (2/510) and 1.2% (6/503), respectively. The prevalence of *NTRK* fusion in CRCs was higher compared with previous studies, which reported a prevalence of 0.23% [17]. These findings could be explained by the fact that our cohort was narrowed down by excluding cases harboring known driver mutations, such as *KRAS*, *NRAS*, and *BRAF* mutations in CRC.

Unfortunately, all nine *NTRK*-rearranged tumors (two NSCLCs, six CRCs, and one IMT) showed no characteristic histological features that could be useful morphological clues for the presence of *NTRK* fusion. 

In this study, five different partner genes were identified. The majority of *NTRK* fusions are *TPM3–NTRK1* rearrangements, which are recurring events in CRCs and are associated with tumor sensitivity to TRKA kinase inhibition [18,19]. As expected, four out of the nine *NTRK* fusions were *TPM3–NTRK1*, followed by *ETV6–NTRK3* (*n* = 2), *LMNA–NTRK1* (*n* = 1), *SQSTM1–NTRK3* (*n* = 1), and *CD74–NTRK1* (*n* = 1). 

Immunoreactivity for TRK was easily identifiable, as the majority of the positive CRC and NSCLC cases showed diffuse moderate to strong intensity staining except for one case harboring *ETV6–NTRK3* fusion. The *ETV6–NTRK3* fusion case showed weak to intermediate nuclear staining. Weak pan-Trk IHC expression was commonly observed in various tumors, including CRCs harboring an *ETV6–NTRK3* fusion, and it was recently demonstrated that the lower sensitivity of pan-Trk IHC was caused by *NTRK3* fusion, especially for *ETV6–NTRK3* fusion [20,21,22,23]. Notably, no false-positive CRC cases were identified in the pan-Trk IHC in this study. These findings are consistent with previous reports that pan-Trk IHC has 100% specificity for CRCs [19,22]. 

The six *NTRK*-rearranged CRCs were all MSI-H tumors, a significantly higher proportion than the 8% proportion of MSI-H in the entire CRC population. As in previous reports [19,20,24], we confirmed once again that *NTRK*-positive CRCs demonstrated a higher frequency of MSI. Therefore, a subset of CRCs harboring no known driver mutations and exhibiting MSI-H should be tested using pan-Trk IHC and further validated using RNA-based targeted NGS.

*NTRK* fusions are highly enriched in several rare specific tumor types, such as secretory carcinomas of the salivary gland and breast, congenital mesoblastic nephroma, pediatric melanoma, and infantile fibrosarcoma [1,4,25]. In our study, efforts to discover *NTRK*-rearranged tumors in tumors known to rarely harbor *NTRK* fusions led to perform the pan-Trk IHC in IMTs. IMT is a distinctive, rarely metastasizing neoplasm composed of myofibroblastic and fibroblastic spindle cells accompanied by an infiltration of lymphoplasma cells [26]. IMTs are genetically heterogeneous, and most of them harbor gene rearrangements of receptor tyrosine kinases, including *ALK* (approximately 50–60%), *ROS1* (approximately 5–10%), and *NTRK3* (approximately 5%) but rarely *RET* and *PDGFRβ* fusion [27,28,29,30,31]. Recent translational studies provided evidence of the potential activity of TKIs in sarcoma, including larotrectenib [32,33,34] 

However, until recently, there have been few analyses to identify *NTRK* fusions in a large number of IMTs. To the best of our knowledge, this study evaluated *NTRK* fusion in the largest number of IMTs. In this study, *NTRK* fusion transcript was identified with *ETV6–NTRK3* in only one IMT (1%). The lower-than-expected frequency may be due to the analysis of IMT samples, including 21 *ALK*-rearranged IMTs.

In only one *NTRK*-rearranged IMT case, immunoreactivity for TRK was heterogeneous and showed moderate nuclear staining intensity that was readily discernible in the less-cellular myxoid area. As shown in a recently published study, although the number of cases was very small, sensitivity and specificity were 100% for IMT [22]. Unfortunately, five false-positive cases were identified in this study, and the cytoplasmic staining pattern in the pan-Trk IHC assay was observed in all the false-positive cases. A limitation of the diagnostic utility of weak and moderate cytoplasmic pan-Trk staining, in contrast to the nuclear staining pattern, was identified. Especially in cases with mesenchymal tumors that show neural and smooth muscle differentiation, the interpretation of pan-Trk IHC should be considered due to physiological cytoplasmic expression of pan-Trk in neural and smooth muscle tissue and their malignant counterparts [14,35,36]. It is also important to note that false-positive results can be caused by the high level of surrounding background staining although the tumor cells themselves were negative. Therefore, several studies have recommended that tumors with neural and smooth muscle differentiation should not be screened using pan-Trk IHC for *NTRK* fusions [37,38]. 

However, our study showed cytoplasmic staining but not nuclear staining in the false-positive IMT cases. Nuclear staining despite focal has been described in tumors harboring *ETV6-NTRK3* fusion protein [22,35,39]. It has also been demonstrated that no nuclear staining was observed in all false-positive cases of sarcoma [22], and pan-Trk nuclear staining is a highly specific diagnostic marker for secretory carcinoma harboring the *ETV6–NTRK3* fusion [40].

Therefore, relevant nuclear staining in a sub-diagnostic manner (focal and weak) but not cytoplasmic staining may be meaningful in mesenchymal tumors when further validated by RNA-based targeted NGS in all advanced and metastatic *ALK* negative-IMTs; this can identify patients who will benefit from TRK inhibitor therapy.

Our study had several limitations. First, our study used TMA-based IHC as a primary screening tool for detecting solid tumors harboring *NTRK* fusions. There may be the possibility of missing positive cases due to heterogeneous staining patterns although recent studies have reported that pan-Trk IHC shows a uniform staining pattern within the same CRC section [19,41]. Another limitation of our study is that the sensitivity and specificity of the pan-Trk IHC assay could not be accurately determined, as RNA-based targeted NGS was not performed in cases with negative IHC results. Finally, this was a retrospective study, and none of our patients received a TRK inhibitor to obtain information regarding treatment response.

The caveats and limitations of pan-Trk IHC for tumor type should be considered when interpreting the IHC results. In IMT, focal, weak cytoplasmic and membranous staining for pan-Trk does not serve as a surrogate marker for *NTRK* fusion, whereas focal, weak nuclear staining indicates the presence of *NTRK* fusion. In NSCLC and CRC, the majority of *NTRK* fusions were readily discernible due to diffuse homogenous moderate to strong cytoplasmic staining on pan-TRK IHC. It should also be noted that focal, weak nuclear staining indicative of *ETV6–NTRK3* fusion should not be overlooked. 

## 5. Conclusions

In conclusion, the utility of pan-Trk IHC should be assessed considering the difference in the performance of pan-Trk IHC depending on the tumor type. Despite its rarity, this study confirms the importance of identifying potential target groups based on the pathological and immunohistochemical characteristics of *NTRK* fusion-driven solid tumors for effective targeted therapy.

## Figures and Tables

**Table 1:** Clinicopathological characteristics of 15 TRK expression cases.

Tumor Type	Case No.	Age at Diagnosis	Sex	Final Diagnosis	MSI Status
Non-small cell lung carcinoma (NSCLC)	L099	68	M	Adenocarcinoma, poorly differentiated (solid pattern)	
	L347 *	42	M	Adenocarcinoma, moderately differentiated (papillary pattern)	
	L491 *	54	F	Adenocarcinoma, moderately differentiated (acinar pattern)	
Colorectal carcinoma (CRC)	C175 *	78	F	Mucinous adenocarcinoma	MSI-high
	C178 *	75	F	Adenocarcinoma, poorly differentiated	MSI-high
	C216 *	73	F	Adenocarcinoma, poorly differentiated	MSI-high
	C421 *	68	F	Adenocarcinoma, moderately differentiated	MSI-high
	C478 *	65	F	Metastatic Adenocarcinoma	MSI-high
	C503 *	60	M	Adenocarcinoma, moderately differentiated	MSI-high
Inflammatory myofibroblastic tumor (IMT)	R016	72	M	Inflammatory myofibroblastic tumor	
	R025 *	41	F	Inflammatory myofibroblastic tumor	
	R042	28	M	Inflammatory myofibroblastic tumor	
	R082	33	M	Inflammatory myofibroblastic tumor	
	R087	5	F	Inflammatory myofibroblastic tumor	
	R093	45	M	Inflammatory myofibroblastic tumor	

* Confirmed cases of *NTRK* gene fusions in NGS assays.

**Table 2:** Pan-Trk immunohistochemical and molecular characteristic of 15 TRK expression cases.

Tumor Type	Case No.	TRK IHC Staining Intensity	TRK IHC Staining Pattern	Fusion Type	Exon # of BP	ChrA	GeneA	Break Point A	ChrB	GeneB	Break Point B	Supporting Reads
Non-small cell lung carcinoma (NSCLC)	L099	1–2	C, M									
	L347 *	2	C, M	*SQSTM1-NTRK3*	S(6)N(14)	chr5	*SQSTM1*	chr5: 179252226	chr15	*NTRK3*	chr15: 88576276	6584
	L491 *	3	C, M	*CD74-NTRK1*	C(7)N(10)	chr5	*CD74*	chr5: 149782684	chr1	*NTRK1*	chr1: 156844362	214
					C(6)N(10)	chr5	*CD74*	chr5: 149784243	chr1	*NTRK1*	chr1: 156844361	1199
Colorectal carcinoma (CRC)	C175 *	3	C, M	*TPM3-NTRK1*	T(7)N(10)	chr1	*TPM3*	chr1: 154142876	chr1	*NTRK1*	chr1: 156844361	318
	C178 *	3	C, M	*TPM3-NTRK1*	T(7)N(10)	chr1	*TPM3*	chr1: 154142876	chr1	*NTRK1*	chr1: 156844361	673
	C216 *	3	C, M	*TPM3-NTRK1*	T(7)N(10)	chr1	*TPM3*	chr1: 154142876	chr1	*NTRK1*	chr1: 156844361	50
	C421 *	1-2	C, M	*TPM3-NTRK1*	T(7)N(10)	chr1	*TPM3*	chr1: 154142876	chr1	*NTRK1*	chr1: 156844361	258
	C478 *	3	NM	*LMNA-NTRK1*	L(13)N(12)	chr1	*LMNA*	chr1: 156108546	chr1	*NTRK1*	chr1: 156845310	9
					L(13)N(12)	chr1	*LMNA*	chr1: 156109604	chr1	*NTRK1*	chr1: 156845310	663
	C503 *	2	N	*ETV6-NTRK3*	E(5)N(15)	chr12	*ETV6*	chr12: 12022900	chr15	*NTRK3*	chr15: 88483984	217
Inflammatory myofibroblastic tumor (IMT)	R016	2	C									
	R025 *	2	N	*ETV6-NTRK3*	E(5)N(15)	chr12	*ETV6*	chr12: 12022900	chr15	*NTRK3*	chr15: 88483984	31
	R042	1–2	C									
	R082	1–2	C									
	R087	1–2	C									
	R093	2	C									

Abbreviation: C, cytoplasmic; M, membranous; N, nuclear; NM, nuclearmembranous; BP, breakpoint. * Confirmed cases of *NTRK* gene fusions in NGS assays.

## Data Availability

Not applicable.

## Abbreviations

NTRK, neurotrophin kinase; TRK, tyrosine receptor kinase; NSCLC, non-small cell lung cancer; CRC, colorectal cancer; IMT, inflammatory myofibroblastic tumor; IHC, immunohistochemistry; NGS, next-generation sequencing.
